# Trigeminal Cardiac Reflex and Cerebral Blood Flow Regulation

**DOI:** 10.3389/fnins.2016.00470

**Published:** 2016-10-20

**Authors:** Dominga Lapi, Rossana Scuri, Antonio Colantuoni

**Affiliations:** ^1^Department of Clinical Medicine and Surgery, “Federico II” University Medical SchoolNaples, Italy; ^2^Department of Translational Research on New Technologies in Medicine and Surgery, University of PisaPisa, Italy

**Keywords:** trigeminal cardiac reflex, mandibular extension, pial arterioles, vasomotion, brain

## Abstract

The stimulation of some facial regions is known to trigger the trigemino-cardiac reflex: the main stimulus is represented by the contact of the face with water. This phenomenon called diving reflex induces a set of reactions in the cardiovascular and respiratory systems occurring in all mammals, especially marine (whales, seals). During the immersion of the face in the water, the main responses are aimed at reducing the oxygen consumption of the organism. Accordingly reduction in heart rate, peripheral vasoconstriction, blood pooling in certain organs, especially the heart, and brain and an increase in blood pressure have been reported. Moreover, the speed and intensity of the reflex is inversely proportional to the temperature of the water: more cold the water, more reactions as described are strong. In the case of deep diving an additional effect, such as blood deviation, has been reported: the blood is sequestered within the lungs, to compensate for the increase in the external pressure, preventing them from collapsing. The trigeminal-cardiac reflex is not just confined to the diving reflex; recently it has been shown that a brief proprioceptive stimulation (10 min) by jaw extension in rats produces interesting effects both at systemic and cerebral levels, reducing the arterial blood pressure, and vasodilating the pial arterioles. The arteriolar dilation is associated with rhythmic diameter changes characterized by an increase in the endothelial activity. Fascinating the stimulation of trigeminal nerve is able to activate the nitric oxide release by vascular endothelial cells. Therefore, the aim of this review was to highlight the effects due to trigeminal cardiac reflex induced by a simple mandibular extension. Opposite effects, such as hypotension, and modulation of cerebral arteriolar tone, were observed, when these responses were compared to those elicited by the diving reflex.

## Introduction

In recent years several clinical studies allowed one to assert that the trigeminal cardiac reflex (TCR) is a clinical phenomenon characterized by hemodynamic perturbations, such as reduction in arterial hypotension, bradycardia, respiratory (apnea), and gastric changes (hypermobility). These effects are triggered by stimulation of any branch of the fifth cranial nerve along its course (Kumada et al., 1975, 1977; Schaller et al., 1999; Schaller, 2004, 2005; Schaller et al., 2009a).

It has been suggested that the TCR is due to sensory nerve endings of the trigeminal nerve. The neuronal signals, originated by these endings, are conducted to the sensory nucleus of the trigeminal nerve, via the Gasserian ganglion, forming the afferent pathway of the reflex arc (White and McRitchie, 1973; Schaller et al., 1999).

This afferent pathway continues along the short internuncial nerve fibers in the reticular formation to connect with the efferent pathway in the motor nucleus of the vagus nerve. Several lines of experimental evidence demonstrate that trigeminally induced cardiovascular reflexes could be mediated initially in the trigeminal nucleus caudalis. Subsequently, the parabrachial nucleus, the rostral ventrolateral medulla oblongata, the dorsal medullary reticular field, and the paratrigeminal nucleus are involved in animal models (White and McRitchie, 1973; Schaller et al., 1999). Because the TCR involves all these structures, it was interesting to evaluate the effects exerted by TCR on the cerebral circulation, because hypotension, and bradycardia could induce cerebral blood flow reduction.

Experimental studies, carried out in rabbits, indicate that TCR represents an expression of a central neurogenic reflex leading to rapid cerebrovascular vasodilation, generated by excitation of oxygen-sensitive neurons in the rostral ventrolateral medulla oblongata (Campbell et al., 1994; Ichinohe et al., 1997; Sires et al., 1998; Schaller, 2004). However, these data have been detected only in the cortex. According to this physiological response, the adjustments of the systemic and cerebral circulations can divert blood to the brain or increase cerebral blood flow.

Several clinical observations indicate that TCR causes changes in cerebral cortex blood flow (White and McRitchie, 1973; Loewinger et al., 1987; Bainton et al., 1990; Kosaka et al., 2000; Burnstine, 2002; Schaller et al., 2007, 2009b). No data, however, have been reported about the influence of TCR on the cerebral blood flow in the short and long term follow up period after the reflex beginning.

## TCR effect's on cerebral blood flow

It is known that TCR occurs during both the peripheral and the central manipulations of the trigeminal nerve (Schaller, 2007). Brunelli et al. showed that a brief proprioceptive stimulation (10 min) by jaw extension causes hypotension and bradycardia in humans (Brunelli et al., 2012).

Successively, Lapi et al. demonstrated that a single mandibular extension (ME), consisting in a submaximal rat mouth opening for 10 min by means of a dilator, is accompanied by an unusually prolonged reduction (about 3 h) of heart rate (HR) and mean arterial blood pressure (MABP) (Lapi et al., 2013, 2014a). This effect was found to be associated with a characteristic and prolonged modulation of cerebral arteriolar tone. At first, vasoconstriction concomitant with the ME stimulus was observed; successively, a prolonged vasodilation occurred, lasting for 3 h, during the entire experimental observation period (Lapi et al., 2013).

Although many aspects related to the underlying mechanisms remain to be explained, some of them are now clear. On one hand, it has been shown that these effects are abolished when the three peripheral trigeminal branches are bilaterally cut, indicating that they may be included among the so-called trigemino-cardiac reflexes (Lapi et al., 2013). These results are in agreement with the studies by Kumada and Schaller who observed that trigeminal nerve stimulations are accompanied by (brief) hypotensive and bradycardic responses in anesthetized rabbits and in humans during neurosurgical intervention, respectively (Kumada et al., 1977; Schaller, 2004; Schaller et al., 2007).

Furthermore, it was observed that these effects depend on the ME stimulation length, i.e., less prolonged and pronounced, when the ME lasted 5 min compared to 10 min stimulation. Finally, it has been shown that the initial brief vasoconstriction response involves an opioid-receptor mediated mechanism, because this response is abolished by the opioid-receptor antagonist naloxone. The subsequent prolonged vasodilation involves a nitric oxide mediated mechanism, because it was blunted by the nitric oxide synthase inhibitor (L-N^G^-Nitroarginine methyl ester: L-NAME) (Lapi et al., 2014b). A subsequent *in vitro* study, carried out by Lapi and coworkers, showed an increase in the neuronal nitric oxide synthase (nNOS) expression during and after ME, unable to result in vasodilation during ME. However, an increase in the endothelial nitric oxide synthase (eNOS) expression, 2 h after ME, was demonstrated.

## TCR effect's on cerebral arteriolar tone modulation

It is interesting to note that the arterioles display rhythmic variations in diameter, termed vasomotion, triggered by smooth muscle cells; however, the endothelium is able to play a modulatory role on vessel tone (Bertuglia et al., 1999). Analyzing the arteriolar diameter changes on 30 min long-term tracings under baseline conditions and after ME, it was possible to enhance the resolution of the low-frequency components, according to Stefanovska's results in humans (Stefanovska et al., 1999). The frequency components around 1.0, 0.3, 0.1, 0.04 Hz have been related to the heart rate, respiration, intrinsic myogenic activity of vascular smooth muscle cells and neurogenic activity on the vessel wall, respectively. Moreover, the frequency component in the range 0.0095–0.021 Hz appears to be modulated by the vascular endothelium (Kvernmo et al., 1999; D'Addio et al., 2013).

**Figure 1:**
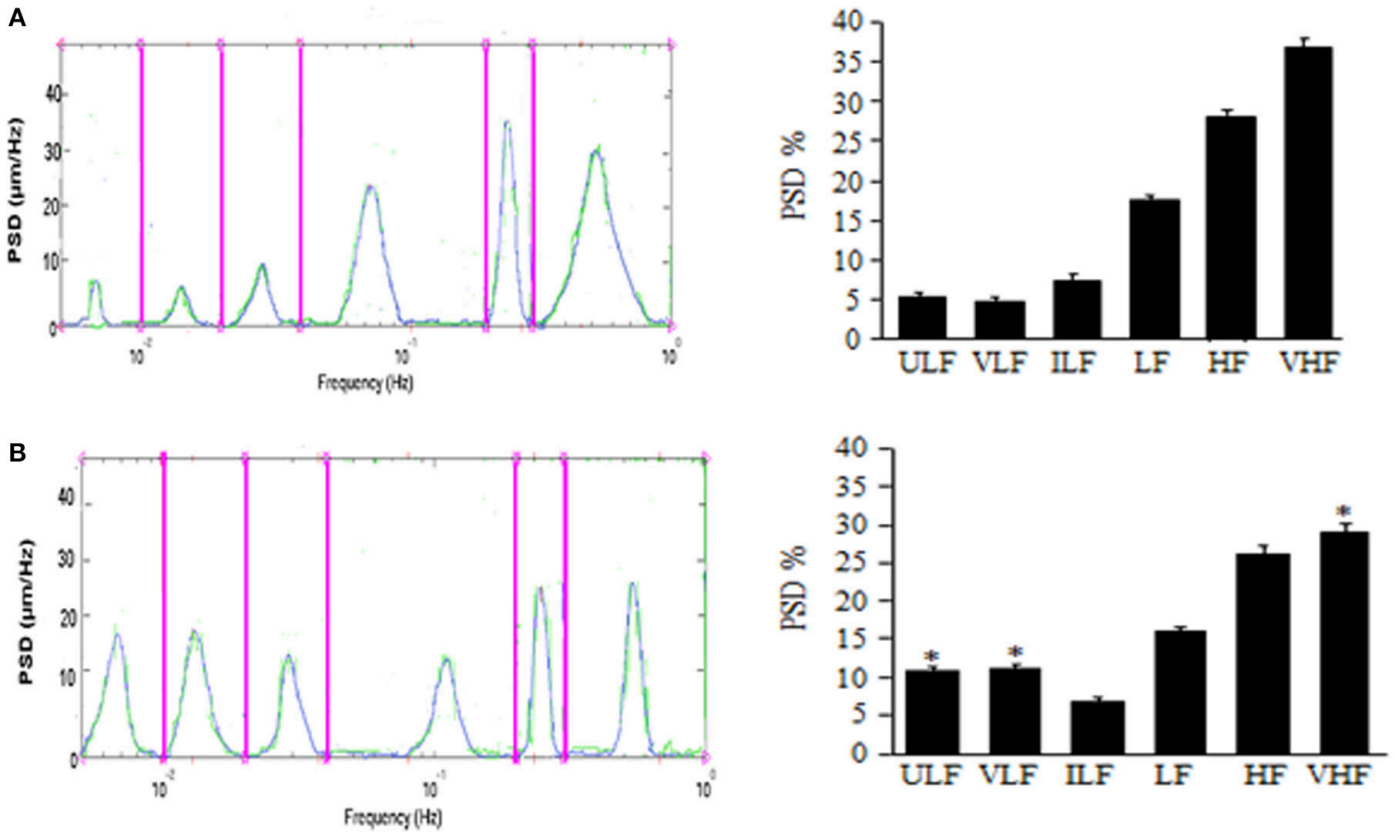
**Effects of mandibular extension on rhythmic diameter changes in pial arterioles (mean diameter: 30.0 ± 2.5 μm)**. Rats subjected to ME, the rhythmic diameter changes (left) and the main corresponding frequency components (right), expressed as percent normalized power spectral density (PSD: μm2/Hz) were measured in baseline conditions **(A)** and during vasodilation **(B)**. ME caused a significant increase of the ULF and VLF frequency components and a decrease of VHF component. ULF, ultra low frequency component; VLF, very low frequency component; ILF, intermediate frequency components; LF, low frequency component; HF, high frequency component; VHF, very high frequency component. ^*^Significantly different from the baseline value.

The results by Lapi et al. demonstrate the pial arterioles undergo rhythmic diameter changes in sham-operated rats. In 30 min recordings six frequency components were detected: (1) ULF (endothelial activity NO-independent) 0.005–0.0095 Hz, (2) VLF (endothelial activity NO-dependent): 0.0095–0.021 Hz, (3) ILF (neurogenic activity): 0.021–0.052 Hz, (4) LF (myogenic activity): 0.052–0.145 Hz, (5) HF (respiratory activity): 0.145–2.00 Hz and (6) VHF (heart beat): 2.500–4.000 Hz (Lapi et al., 2014a). This pattern was detected during the whole observation time (over 3 h), while MABP (125 ± 10 mmHg) and HR (330 ± 25 bpm) remained unchanged (Figure 1A).

In rats submitted to 10 min ME, under baseline conditions MABP was 130 ± 15 mmHg, HR was 325 ± 30 bpm (*p* = NS vs. sham operated rats) and rhythmic arteriolar oscillations were not different compared to those observed in sham operated rats (Lapi et al., 2014a). After ME, MAP, and HR decreased by 20.5 ± 1.2 and 23.0 ± 0.8% of baseline, respectively; conversely, the amplitude of the first and second frequency components increased: ULF by 7.0 ± 1.5% and VHF by 8.0 ± 2.0% of baseline (Figure 1B). Concomitantly, the other frequency components decreased compared with those detected under baseline conditions. These effects lasted up to 3 h after ME (Lapi et al., 2013).

Therefore, a brief (10 min) and passive ME causes a significant and prolonged decrease in MABP and HR, accompanied by an increase in pial arteriolar diameter according to previously reported data (Lapi et al., 2013). Vasodilation is characterized by an increase in the spectral density of the lowest frequencies related to endothelial activity, ULF and VLF (ranges: 0.005–0.0095 Hz and 0.0095–0.021 Hz, respectively).

ME appears to affect the mechanisms involved in the regulation of pial arteriolar tone for long time, likely facilitating the perfusion of cerebral tissue through a modulation of the rhythmic arteriolar diameter changes.

Up to day, the mechanisms whereby ME is accompanied by a prolonged reduction in blood pressure and heart rate remain uncertain. However, ME-induced hypotension may fall into the category of the so-called trigemino-cardiac reflexes extensively reviewed by Schaller (Schaller, 2007). TCR has been proposed to represent the expression of a neuroprotective central neurogenic reflex leading to rapid cerebrovascular vasodilatation in response to facial and nasal mucosal stimulation, such as during diving. Moreover, the reflex may be of potential relevance in brain injury states (Schaller, 2004). Finally, TCR induced by ME as well as the systemic responses, related to activation of vagal efferent discharge, affect the mechanisms involved in the regulation of pial arteriolar tone, increasing the endothelial activity, and facilitating the redistribution of cerebral blood flow supply.

In conclusion, these deep insights into cerebral hemodynamics changes during TCR could stimulate clinical trials and could suggest the development of suitable non-invasive devices, useful in the treatment of disease with impaired cerebral arteriolar tone.

## Author contributions

DL: substantial contributions to the conception, analysis and interpretation of data for the work. DL, RS, AC: revising it critically for important intellectual content. AC: Final approval of the version to be published.

### Conflict of interest statement

The authors declare that the research was conducted in the absence of any commercial or financial relationships that could be construed as a potential conflict of interest.
